# Proximal screws placement in intertrochanteric fractures treated with external fixation: comparison of two different techniques

**DOI:** 10.1186/1749-799X-6-48

**Published:** 2011-09-22

**Authors:** Marios D Vekris, Marios G Lykissas, Gregory Manoudis, Alexandros N Mavrodontidis, Christos D Papageorgiou, Anastasios V Korompilias, Ioannis P Kostas-Agnantis, Alexandros E Beris

**Affiliations:** 1 Department of Orthopaedic Surgery, University of Ioannina School of Medicine, Ioannina, Greece

**Keywords:** Intertrochanteric fractures, Pertrochanteric fixator, Harris Hip Score, Parker mobility score

## Abstract

**Background:**

To compare two different techniques of proximal pin placement for the treatment of intertrochanteric fractures in elderly patients utilizing the Orthofix Pertrochanteric Fixator.

**Methods:**

Seventy elderly high-risk patients with an average age of 81 years were treated surgically for intertrochanteric fracture, resulting from a low energy trauma. Patients were randomly divided in two groups regarding to the proximal pin placement technique. In Group A the proximal pins were inserted in a convergent way, while in Group B were inserted in parallel.

**Results:**

All fractures healed uneventfully after a mean time of 98 days. The fixator was well accepted and no patient had significant difficulties while sitting or lying. The mean VAS score was 5.4 in group A and 5.7 in group B. At 12 months after surgery, in group A the average Harris Hip Score and the Palmer and Parker mobility score was 67 and 5.8, respectively. In group B, the average Harris Hip Score and the Palmer and Parker mobility score was 62 and 5.6, respectively. No statistically significant difference was found regarding the functional outcome. The mean radiographic exposure during pin insertion in Group A and Group B was 15 and 6 seconds, respectively. The difference between the two groups, regarding the radiographic exposure, was found to be significant.

**Conclusion:**

Proximal screw placement in a parallel way is simple, with significant less radiation exposure and shorter intraoperative duration. In addition, fixation stability is equal compared to convergent pin placement.

## Background

Hip fractures are a leading cause of disability among the elderly. Treatment goals for this patient population include early mobilization with restoration of the anatomic alignment of the proximal part of the femur and maintenance of the fracture reduction. During the 1950's external fixation was introduced for the management of intertrochanteric fractures.1 Although the first reports were promising, a high prevalence of postoperative complications such as pin-loosening, infection, and mechanical failure of the fixator resulted in discontinuation of its use [1]. The development of external fixators and the introduction of new materials such as the hydroxyapatite-coated pins prompted surgeons to reconsider external fixator as an alternative method for the treatment of intertrochanteric fractures in elderly high-risk patients [2-4].

This prospective randomized study aimed to present our experience in treating intertrochanteric fractures in elderly patients using the pertrochanteric external fixator and compare two different techniques of proximal pin placement in terms of functional outcome, procedure simplicity and radiation exposure.

## Methods

**Figure 1:**
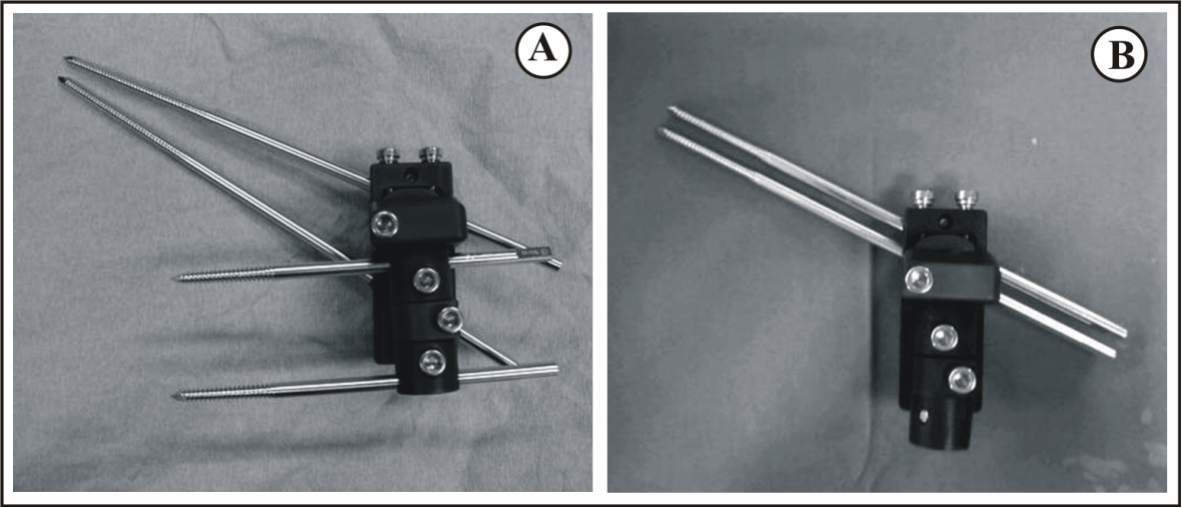
**Proximal screws placement**. The Orthofix Pertrochanteric Fixator (Pertrochanteric Fixator, Orthofix, Verona, Italy) with the proximal screws placed in convergent **(A) **or parallel **(B) **way.

The study design was approved by the ethics committee. Seventy patients, 25 men and 45 women, with an average age of 81 years (range; 69-96 years) were treated surgically for intertrochanteric fracture, resulting from a low energy trauma. The Orthofix Pertrochanteric Fixator (Pertrochanteric Fixator, Orthofix, Verona, Italy) was utilized in all cases (Figure 1). This device offers additional theoretical advantages, such as simplicity and versatility in pin placement, improved stability due to the rigid frame, and minimal stiffness of the ipsilateral knee joint due to the small size of the device.

All of our patients were elderly high-risk patients with several comorbidities such as heart failure, coronal artery disease, hypertension, renal failure, malignancy, high level of thyroid hormones, anemia, or pulmonary disease (Table 1). Osteoporosis was also present in all patients. Exclusion criteria included dementia, reverse obliquity fractures, previous hip fracture, and pathological fractures. Patients with diabetes mellitus were not treated with external fixation due to the increased risk of pin-track infection. This patient population was randomly placed in two groups regarding to the proximal pin placement technique. In the first group (Group A; n = 35) the proximal pins were inserted in a convergent way, as proposed by the manufacturer, while in the second group (Group B; n = 35) the proximal pins were inserted in parallel, which is our modification of the technique.

**Table 1:** Concomitant diseases in patients with intertrochanteric fractures of the hip treated with external fixation

Concomitant diseases	n
**Heart failure**	39
**Coronal disease**	32
**Hypertension**	48
**Renal disease**	16
**Thyreoeidopathy**	5
**Anemia**	15
**Pulmonary disease**	22
**Malignancy**	1

According to the American Society of Anesthesiologists, 47 patients were scored as ASA 3 and 23 patients as ASA 4. In group A, 12 patients had an AO type A1 fracture and 23 patients had an AO type A2 fracture. In group B, 13 patients had an AO type A1 fracture and 22 patients had an AO type A2 fracture. Before surgery no significant difference was noted between the two Groups regarding the fracture type. All patients were operated within the first three days after admission (mean; 2 days).

### Surgical Technique

Fifty-three patients had spinal anesthesia whereas 17 patients had general anesthesia. With the patient in a supine position on a fracture table, holding the leg under controlled traction the fracture was reduced in both planes under image intensification. Fracture reduction was assessed by evaluating major fragment translation and the femoral neck-shaft angle. Less than 5 mm of translation or gap and a neck-shaft angle with minor valgus (< 15 degrees) compared with the other leg were considered as a sufficient reduction on the anteroposterior view. In the lateral view less than 20 degrees of angulation was considered acceptable [5].

Under fluoroscopic control two proximal and two distal 6.5-mm self-drilling and self-tapping screws were percutaneously inserted along the femoral neck and into the proximal femoral shaft, respectively.

**Figure 2:**
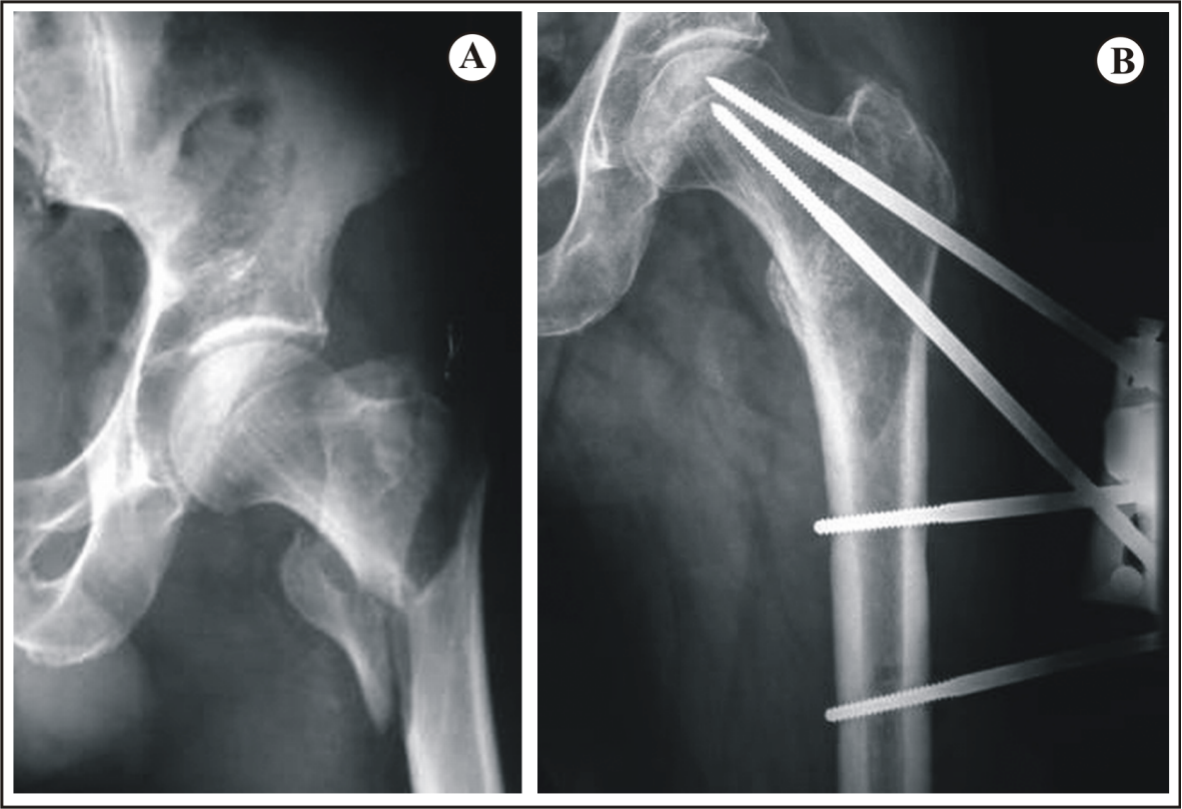
**Clinical case with proximal screws placed in a convergent way**. **A**. Anteroposterior view of an AO Type II intertrochanteric fracture in an 82-year old man. **B**. Anteroposterior view following fixation of the fracture with the fixator applied in a satisfactory position and the proximal screws placed in a convergent way.

In Group A, the most proximal screw was inserted first along the femoral neck within 5 mm from the superior cortex. The second proximal screw was inserted in a slight convergent way according to the Orthofix operative technique passing near the medial cortex (Figure 2) [6].

**Figure 3:**
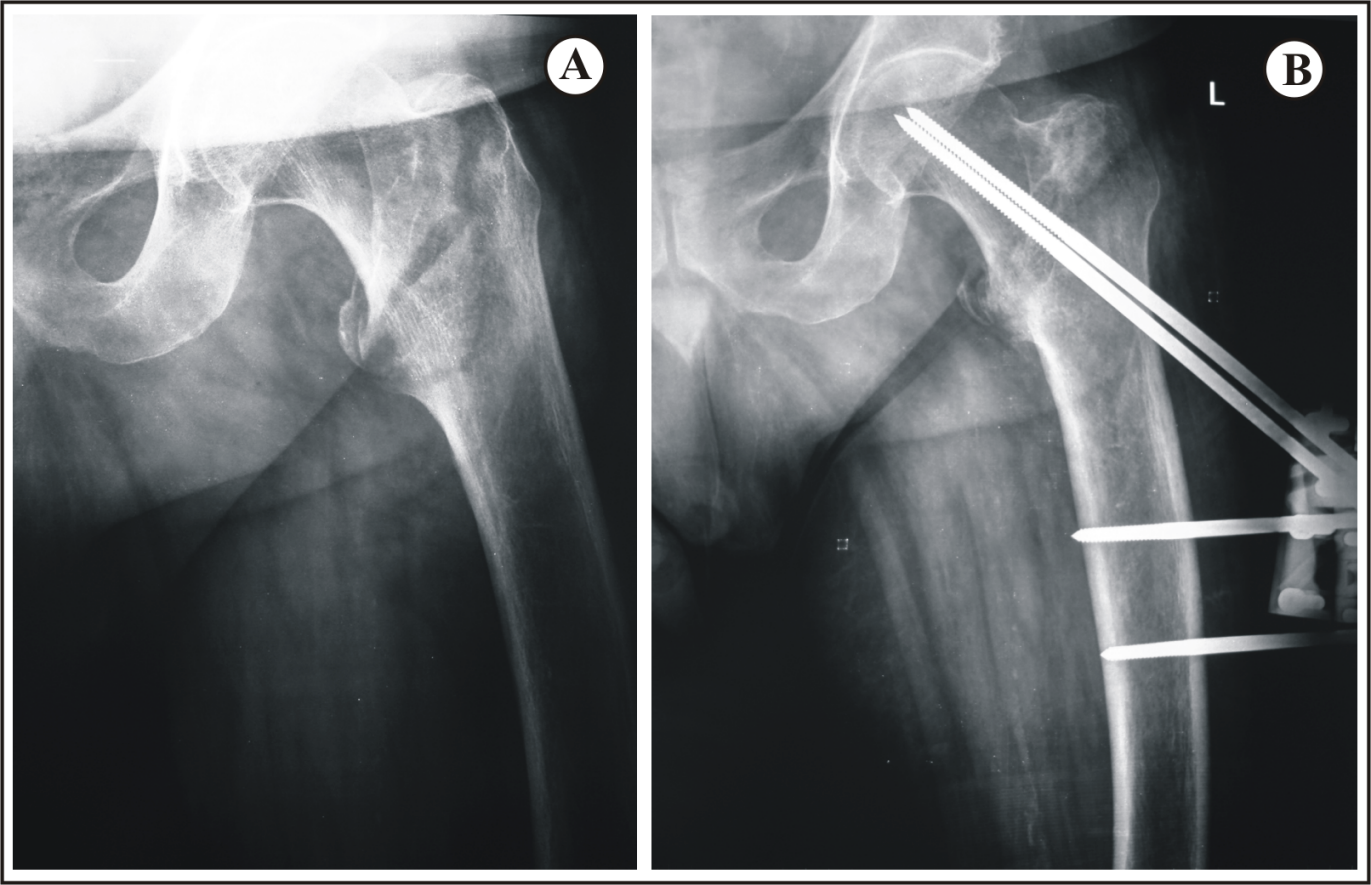
**Clinical case with proximal screws placed in a parallel way**. **A**. Anteroposterior view of an AO Type I intertrochanteric fracture in an 75-year old man. **B**. Anteroposterior view following fixation of the fracture with the proximal screws placed in a parallel way.

In Group B, a 2-mm Kirschner-wire was inserted along the femoral neck as proximal as possible to the medial cortex and at the center of the femoral neck in the lateral view. The appropriate position of the K-wire was confirmed by fluoroscopy at this point. Attention was paid to ensure that the tip of the screw was at least 5 mm far from the articular surface to prevent penetration. Using a special screw guide the first screw was inserted parallel to the K-wire, taking care to place it as near as possible to the medial cortex and at the center of the femoral neck by rotating the screw guide around the K-wire axis. The second screw was inserted parallel to the first one, following the screw seat of the external fixator proximal clamp and in the same depth as the first screw, requiring no extra radiation exposure (Figure 3). Moreover, it was possible to place the second screw slightly anteriorly or posteriorly in the femoral neck, by minimally rotating the device, if there was anterior or posterior cortex comminution. This modification leads to accurate proximal pin placement with minimal radiation exposure.

The two distal screws were inserted perpendicular to the long axis of the proximal femoral shaft and were implanted to a depth of two screw threads beyond the opposite bone cortex. In cases of subtrochanteric extension of the fracture, the posterior clamp of the device was rotated through 180 degrees allowing more distal screw placement.

In 12 patients with comminuted medial cortex fractures, 5 from group A and 7 from group B, demineralized bone matrix allograft was injected through a small incision over the greater trochanter.

The time of radiation exposure was measured in both groups.

### Postoperative Management

Evaluation during treatment included plain radiographs and pain assessment using the visual analog scale (VAS). Clinical evaluation of patients was assessed with the Harris Hip Score [7] and the Palmer and Parker mobility score [8] at six months after surgery. Preoperative walking ability and residential accommodation were also recorded (Table 2).

**Table 2:** Pre- and postoperative walking ability

	Independent	Walking stick	Two sticks	Walking frame	Inability
**Pre-fracture**	32	27	5	5	1
**Post-fracture**	19	26	9	8	4

On the first postoperative day, patients were mobilized, sitting on bed or on a chair, while on the second postoperative day partial weight-bearing with a walker or crutches was encouraged. The patients were advised to do partial weight-bearing depending on tolerance to pain. Weight-bearing was gradually increased and full weight-bearing was allowed when clinical and radiological signs of fracture union were present.

Pin entry sites were cleaned with saline solution every two days. Low molecular weight heparin was also administered for deep vein thrombosis prevention.

### Statistical Analysis

Statistical analyses were carried out using SPSS (SPSS statistic package, version 16.0; SPSS Inc., Chicago, IL) statistical software. Mann-Whitney U test was used to determine whether there were any significant differences. The level of significance was set at p < 0.05.

## Results

The average intraoperative time was 25 minutes for group A and 20 minutes for group B (p > 0.05), while the average preparation time was 15 minutes in both groups. The mean radiation exposure during pin insertion in Group A and Group B was 15 and 6 seconds, respectively. This difference was found to be statistically significant (p < 0.05). Intraoperative blood loss was minimal and postoperative haemoglobin levels were similar to the preoperative levels. Four patients with preoperative low hemoglobin levels required blood transfusion within the first 2 postoperative days to facilitate mobilization.

**Figure 4:**
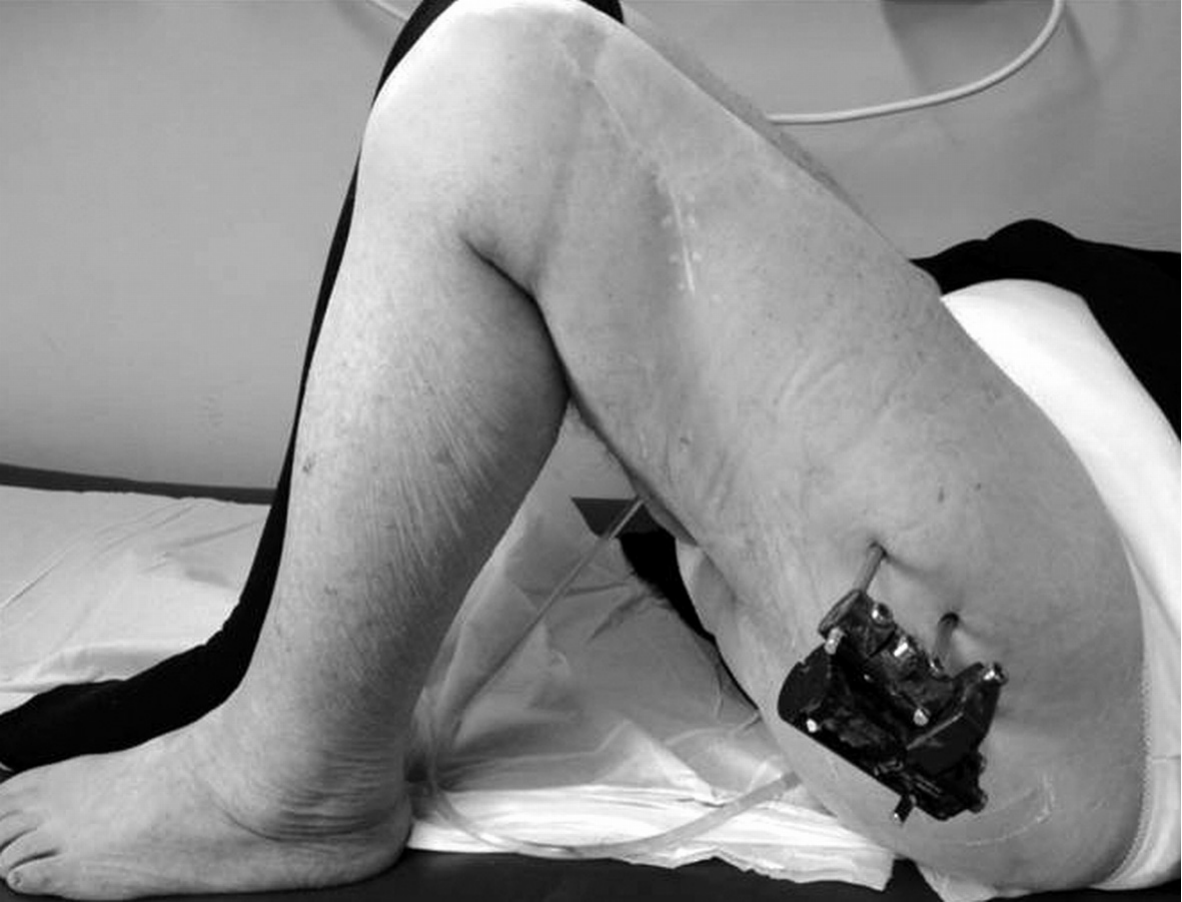
**Postoperative range of motion**. No clinically significant limitation of hip or knee range of motion was observed in patients of either group.

**Figure 5:**
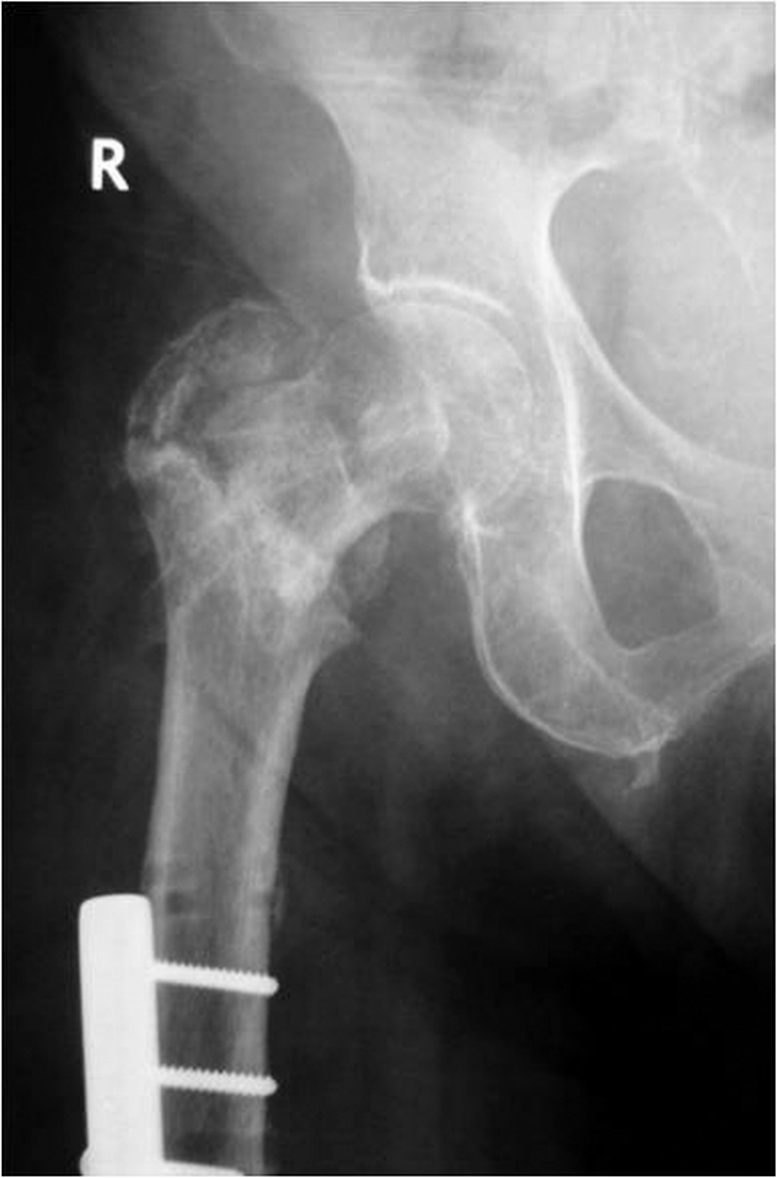
**Complications**. Re-fracture occurred after external fixator removal in one patient 15 weeks after surgery.

Follow-up visits were scheduled at 45, 90, and 180 days after surgery and new x-rays were performed at that time. During a minimum of 12-month follow-up period 6 patients, 2 from group A and 4 from group B, died from causes unrelated to the fracture. In the remaining 64 patients no clinically significant limitation of hip or knee range of motion was observed in patients of either group (Figure 4). External fixator was removed in the outpatient department with local anesthesia after radiological confirmation of fracture consolidation, in a mean time of 98 days (range; 90-120 days) after surgery. Radiographic union was defined by the presence of trabeculae bridging the fracture site or obvious periosteal callus within the fracture line [9]. The more prolonged healing time was noticed in fractures with subtrochanteric extension and medial cortex comminution. All fractures healed uneventfully (100% consolidation rate) in both groups. There was no sign of osteolysis around the screws, neither cut-out of the pins. A re-fracture occurred after external fixator removal in one patient from group A. In this patient, the fracture had not healed at the time of fixator removal. Due to the patient's impaired health, re-fracture was treated non-surgically (Figure 5).

Reduction was considered anatomical when the neck-shaft angle was restored and there was no obvious gap in the fracture line. Forty-eight fractures (68.57%) were anatomically reduced whereas 22 fractures (31.43%) were reduced with valgus angulation of less than 15 degrees or with a small gap or translation of less than 5 mm. Furthermore, the femoral neck-shaft angle and the distance between the femoral head and the tip of the screws on the radiographs obtained prior to device removal were compared with those measured on the immediate postoperative radiographs. In 1 patient of group A and in 2 patients of group B the reduction was lost and the neck-shaft angle was 11 degrees varus on average (range; 10-13 degrees) compared with the immediate postoperative radiographs (p > 0.05). In 4 patients (6.2%), 2 in group A and 2 in group B, migration of less than 5 mm of proximal screws into the femoral head was noticed, but without penetration into the hip joint.

Rehabilitation was directly related to preoperative walking ability and degree of postoperative pain (Table 2). The fixator was well accepted and no patient had significant difficulties while sitting or lying. The mean VAS score was 5.4 (range; 3-9) in group A and 5.7 in group B (range; 3-9) (p > 0.05). At 6 months after surgery, in group A the average Harris Hip Score and the Palmer and Parker mobility score was 67 (range; 46-90) and 5.8, respectively (Table 3). In group B the average Harris Hip Score and the Palmer and Parker mobility score was 62 (range; 43-91) and 5.6, respectively. The difference between Groups A and B was statistically insignificant for both Harris Hip Score and Palmer and Parker mobility score.

**Table 3:** Patients' classification according to Palmer and Parker mobility score

	No problem	With aids	With help from another person	Unable to perform
**Able to get about the house**	3	2	1	0
**Able to get out of the house**	3	2	1	0
**Able to go shopping**	3	2	1	0

In all patients, duration of hospitalization ranged between 4 and 10 days with a mean of 7.3 days. After discharge, 23 patients were moved to a geriatric institution requiring further nursing. Only 7 patients were accommodated in geriatric homes before fracture (Table 4).

**Table 4:** Patients required further nursing in a geriatric institution

	Own home	Geriatric home
**Pre-fracture**	63	7
**Discharge**	47	23
**At final follow-up**	48	16

Pin track infection was developed in 6 patients (9.3%) postoperatively, 2 in group A and 4 in group B. Pin track infection was superficial, located in all patients at the site of insertion of the proximal pins and was treated with broad spectrum oral antibiotics for one week and attentive care of the pin entry points. Six patients, 3 from each group, had bedsore due to prolonged lying. Postoperative complications included pneumonia in 1 patient (1.4%), urinary tract infection in 3 patients (4.2%), and pulmonary embolism in 2 patients (2.8%).

## Discussion

Intertrochanteric hip fractures account for approximately half of all hip fractures in the elderly population. Among these fractures, 50 to 60% are classified as unstable [5,10]. Unstable intertrochanteric fractures occur more often with increased age and low bone mineral density and are associated with a high rate of complications [5,11].

Several methods of fixation have been proposed for the management of intertrochanteric fractures, such as compression hip screw and side plate, dynamic compression sliding plate, fixed angle blade plate, intramedullary sliding hip screw, and lately external fixator [12].

Scott1 first described a method of treating intertrochanteric fractures by skeletal pinning and external fixation. Since then several authors have proposed multiple type of external fixators, but results were not so encouraging [1,13,14]. On the contrary, recent evidence supports that pertrochanteric fractures treated with newly developed external fixators have better results than those reported in previous studies of external fixation [3,15-17]. According to the same authors, external fixation can provide results that are similar to, or even better than, the results obtained with conventional internal fixation techniques. All these studies reported the advantages of external fixation including quick and simple application, minimal blood loss, less radiation exposure, pain reduction, satisfactory stability, and early weight-bearing. Pertrochanteric external fixator has been mainly used in elderly high-risk patients [13,14,18], as well as in multiple injured patients with complex fractures of the subtrochanteric region [19,20].

The authors had the experience with the application of pertrochanteric external fixator. This study was designed in order to establish an easier method of application by minimizing the radiation exposure and the overall surgical time. The average intraoperative time was higher in group A, although no statistically different from the intraoperative time in group B. On the other hand, statistically significant difference was found in radiation exposure between the 2 Groups, with Group B requiring less C-arm usage than Group A for pin insertion.

The present study also confirms the advantages of external fixation for treating intertrochanteric fractures in elderly, high-risk patients. In accordance with previous studies, the mean intraoperative time for application of the fixator was short (21.8 minutes) compared with the one reported in other surgical methods, such as sliding hip screw, dynamic hip screw, intramedullary sliding hip screw, and Enders nails [21-24]. There was no need of blood transfusion since blood loss during surgery was insignificant in opposition to other surgical methods [21,25-27]. These parameters were crucial given that our group consisted of high-risk patients with several co-morbidities. An additional advantage of external fixation was the possibility of application under local anesthesia for patients who have poor general health in whom other options were not applicable [13,20].

Varisation and limp shortening due to varous collapse are mechanical complications commonly reported after either internal or external fixation of unstable or severely osteoporotic intertrohanteric fractures. Although most of the patients in our series had poor bone quality, low incidence of mechanical complications was recorded and was similar in both groups. Varisation of a mean of 11 degrees was noted in 3 cases (4.7%). Migration of the proximal screws into the femoral head was recorded in 4 patients (6.2%). In all cases, the migration was less than 5 mm compared with the initial radiographs, without penetration into the joint or cut out. Vossinakis et al. [17] reported statistically significant lower incidence of proximal screw migration with the external fixator when compared with the sliding hip screw. In cases of proximal screw protrusion into the joint space or cut-out, treatment includes retraction of the offending screw without anesthesia. In our series we did not had any proximal screw migration of more than 5 mm and, more important, no cut out of the superior cortex.

All fractures healed uneventfully in both groups and none of our patients required further operation. Immediate postoperative full loading or lack of control of loading, often seen in elderly people, is usually the cause of reduction loss immediate postoperatively. Comminuted and severely osteoporotic fractures are also prone to lose of initial reduction. Moroni et al. [3], in a similar study by using hydroxyapatite-coated screws, reported bone ingrowth into the coating and lower rate of varus collapse. Therefore, someone may suggest that the use of hydroxyapatite-coated screws could increase the stability of fixation. Furthermore, in stable intertrochanteric fractures the external fixator may act as a tension band [20]. Lateral placement increase the lever arm of the power and augments the physiological stress-reducing effect of the iliotibial tract [20]. In unstable fractures, due to its elasticity, external fixator enhances rapid and exuberant callus formation. Load sharing between the fractured bone and the external fixation is usually achieved and damaging stresses on the fixator are reduced [20]. Large contact surface between the pins and the bone and a degree of controlled sliding that allows slight impaction at the fracture site contribute to mechanical stability as well [28].

Both methods of proximal screw placement showed comparable results. Parallel positioning of the proximal screws however, seems to be simpler method with less radiation exposure of the surgeon. This is due to the simplicity of the second screw placement parallel to the first one using the screw guide that minimizes the use of the C-arm.

In a previous study, Vossinakis et al. [16] proposed parallel insertion of proximal screws, whereas in a most recent study the same authors described convergent positioning of the proximal screws [17]. In both studies adequate results were reported. In our series, positioning of the screws in either parallel or convergent way did not affect the final outcome.

## Conclusion

Our study shows that external fixation is an effective treatment for intertrochanteric fractures in elderly high-risk patients. Operative time is short, blood loss is negligible, and stable fixation permits early mobilization. Proximal screw placement in either parallel or convergent way shows similar results and does not affect the final outcome. However, screw placement in a parallel way is a simpler method with less radiation exposure providing adequate fixation stability and therefore is recommended by the authors.

## Competing interests

There are no competing interests; this is a basic academic research initiative.

## Authors' contributions

All authors contributed equally to this work. MDV, MGL and GM participated in the design of the study and drafted the manuscript. ANM and CDP performed the statistical analysis. AEB, INKA, and AVK participated in its design and coordination and helped to draft the manuscript MDV has had the main responsibility for the study and manuscript preparation. All authors read and approved the final manuscript.

## References

[B1] Scott IH Treatment of intertrochanteric fractures by skeletal pinning and external fixation Clin Orthop Relat Res 1957 10 326 34 13561572

[B2] Magyar G Toksvig-Larsen S Moroni A Hydroxyapatite coating of threaded pins enhances fixation J Bone Joint Surg Br 1997 79 487 9 10.1302/0301-620X.79B3.7190 9180334

[B3] Moroni A Faldini C Pegreffi F Hoang-Kim A Vannini F Giannini S Dynamic hip screw compared with external fixation for treatment of osteoporotic pertrochanteric fractures. A prospective, randomized study J Bone Joint Surg Am 2005 87 753 9 10.2106/JBJS.D.01789 15805203

[B4] Moroni A Heikkila J Magyar G Toksvig-Larsen S Giannini S Fixation strength and pin tract infection of hydroxyapatite-coated tapered pins Clin Orthop Relat Res 2001 388 209 17 11451122 10.1097/00003086-200107000-00029

[B5] Baumgaertner MR Curtin SL Lindskog DM Keggi JM The value of the tipapex distance in predicting failure of fixation of peritrochanteric fractures of the hip J Bone Joint Surg Am 1995 77 1058 64 7608228 10.2106/00004623-199507000-00012

[B6] The Orthofix Pertrochanteric Fixator. Operative technique 1998 Verona: Orthofix Srl

[B7] Harris WH Traumatic arthritis of the hip after dislocation and acetabular fractures: treatment by mold arthroplasty. An end-result study using a new method of result evaluation J Bone Joint Surg Am 1969 51 737 55 5783851

[B8] Parker MJ Palmer CR A new mobility score for predicting mortality after hip fracture J Bone Joint Surg Br 1993 75 797 8 8376443 10.1302/0301-620X.75B5.8376443

[B9] Parker MJ Cutting-out of the dynamic hip screw related to its position J Bone Joint Surg Br 1992 74 625 1624529 10.1302/0301-620X.74B4.1624529

[B10] Koval KJ Aharonoff GB Rokito AS Lyon T Zuckerman JD Patients with femoral neck and intertrochanteric fractures: Are they the same? Clin Orthop Relat Res 1996 330 166 72 8804287 10.1097/00003086-199609000-00020

[B11] Cole P Bhandari M What's new in orthopaedic trauma J Bone Joint Surg Am 2006 88 2545 61 10.2106/JBJS.F.01118 17079430

[B12] Lindskog DM Baumgaertner MR Unstable intertrochanteric hip fractures in the elderly J Am Acad Orthop Surg 2004 12 179 90 15161171 10.5435/00124635-200405000-00006

[B13] Gotfried Y Frish E Mendes DG Roffman M Intertrochanteric fractures in high risk geriatric patients treated by external fixation Orthopedics 1985 8 769 74 4095006 10.3928/0147-7447-19850601-13

[B14] Kamble KT Murthy BS Pal V Rao KS External fixation in unstable intertrochanteric fractures of femur Injury 1996 27 139 42 10.1016/0020-1383(95)00172-7 8730390

[B15] Christodoulou NA Sdrenias CV External fixation of select intertrochanteric fractures with single hip screw Clin Orthop Relat Res 2000 381 204 11 11127657 10.1097/00003086-200012000-00024

[B16] Vossinakis IC Badras LS Management of pertrochanteric fractures in high-risk patients with an external fixation Int Orthop 2001 25 219 22 10.1007/s002640100238 11561494 PMC3620822

[B17] Vossinakis IC Badras LS The external fixator compared with the sliding hip screw for pertrochanteric fractures of the femur J Bone Joint Surg Br 2002 84 23 9 10.1302/0301-620X.84B1.12034 11837827

[B18] Badras L Skretas E Vayanos ED The use of external fixation in the treatment of trochanteric fractures Rev Chir Orthop 1997 83 461 5 9452799

[B19] Buckley JR Caiach SM External fixation in comminuted upper femoral fractures Injury 1993 24 476 8 10.1016/0020-1383(93)90154-X 8406769

[B20] Dhal A Singh SS Biological fixation of subtrochanteric fractures by external fixation Injury 1996 27 723 31 10.1016/S0020-1383(96)00116-7 9135753

[B21] Bridle SH Patel AD Bircher M Calvert PT Fixation of intertrochanteric fractures of the femur J Bone Joint Surg Br 1991 73 330 4 2005167 10.1302/0301-620X.73B2.2005167

[B22] Fornander P Thorngren KG Tornqvist H Ahrengart L Lindgren U Swedish experience with the Gamma nail versus sliding hip screw in 209 randomised cases Int J Orthop Traumatol 1994 4 118 22

[B23] Grosse A Taglalang G Gamma locking nail: surgical technique 1992 London: Howmedica

[B24] Nungu S Orerud C Rehnberg L Treatment of intertrochanteric fractures: comparison of Ender nails and sliding screw plates J Orthop Trauma 1991 5 452 7 10.1097/00005131-199112000-00011 1762007

[B25] Friedmann BA An analysis of surgical blood use in US hospitals with application to the surgical blood order schedule Transfusion 1979 19 268 78 10.1046/j.1537-2995.1979.19379204208.x 452067

[B26] Hardy DC Descamps PY Krallis P Fabeck L Smets P Bertens CL Delince PE Use of an intramedullary hip-screw compared with a compression hip-screw with a plate for intertrochanteric femoral fractures. A prospective, randomized study of one hundred patients J Bone Joint Surg Am 1998 80 618 30 9611022 10.2106/00004623-199805000-00002

[B27] Mac Bride DJ Stother JG Blood transfusion requirements in elderly patients with surgically treated fractures of the femoral neck J Royal Coll Surg Edinberg 1988 33 311 3 3244136

[B28] Scarante B Ranellucci M Lavini F The dynamic axial fixator in the treatment of pertrochanteric fractures of the femur Int J Orthop Traum 1993 3 Suppl 3 58 60

